# Recruiting Duties of the Territorial Regimental Surgeon

**Published:** 1909-05-15

**Authors:** 


### The Royal Army Medical Corps Section.

### RECRUITING DUTIES OF THE TERRITORIAL REGIMENTAL SURGEON.

As the examination of all the recruits of his battalion is one of the duties of the Territorial Regimental Surgeon, and as the War Office Memorandum of March 22, 1909, on the training of the Territorial Medical Service, an outline of which has already been given in The Hospital, specially lays it down that attention is to be paid to the practical instruction of medical officers in this work, it may not be out of place to outline briefly the procedure insisted on by the military authorities. Before doing so, it may be mentioned that paragraph 611 of the Territorial Regulations grants County Associations a fee of 1s. for the medical examination of each recruit who joins the Territorial Force within its county area, the grant being calculated on the number of recruits enlisted during the year ending October 31. This grant is handed over to the regimental surgeon; but it should be noted that the fee is only given for recruits who have been pronounced fit for enlistment. Nothing is allowed for those rejected, although the time of the medical officer is equally expended on them.

This scarcely seems a fair arrangement. In our opinion every recruit examined should be paid for. It will be found that the chief recruiting periods vary in different parts of the country, but with the majority of the Territorial units the early months of the year are the best, so that it is at this time regimental medical officers should calculate on their services being required, and it should be borne in mind that the work may be much facilitated by arranging with the adjutant of the battalion that the recruits will assemble in batches on certain evenings for examination.

The guide to be followed by the regimental surgeon in examining recruits is the pamphlet issued by the military authorities at the end of last year, and entitled "Instructions for the Physical Examination of Candidates for the Territorial Force." Its opening paragraph states definitely the conditions that call for rejection. With these the medical officer must make himself thoroughly acquainted. The list is a long one, but, summarised, it amounts to this, that the object of the medical examination of the Territorial recruit is to testify to the man's *general fitness for service*; in other words, to show that his heart and lungs are sound, that he has no organic disease, that he is free from any serious bodily defect, such as hernia or varicose veins, that his sight and hearing are good, and that he has sufficient number of teeth for the proper mastication of his food. The subsequent paragraphs of the pamphlet enumerate for the guidance of the examiner the order in which the examination is to be carried out, and in its main outline it follows, as far as possible, that adopted in the enlistment of recruits for the Regular Army; but the Territorial regimental surgeon will be wise if he conducts his examination with every consideration for the feelings of the civilian soldier, seeing that as much privacy as possible is given, and that the question of removing all clothing at the same time is not pressed. Removal of portions of clothing at one time, as is done in examination for life assurance, meets all the requirements of the examination.

Of the two documents given to the Territorial recruit on presenting himself for enlistment, one is an attestation form, and on the second page of it will be found a form for the Medical Inspection Report. After the different items of it have been filled in by the medical examiner, he will find underneath it a form of certificate which he has to sign, saying whether or not he considers the recruit fit for admission to the Territorial Force.

The order of examination for completing the Inspection Report commences with taking the height. This should be done with the boots off, the heels being together and touching the standard so as to be sure both heels are on the ground all the time. Should the recruit be measured with his boots on, one inch should be taken off the height. There is no entry for weight, but it should be taken, if possible, so as to serve as a criterion for comparison with the height. Then follows the chest measurement, the maximum on full inspiration being first noted, and then the minimum on full expiration, so as to obtain the range of expansion. For taking the chest measurements the hands should be raised above the head to allow of the tape being adjusted. When in position its posterior upper edge should touch the inferior angles of the shoulder blades, and its anterior lower edge the upper part of the nipples. When the tape is properly adjusted, bring the arms down so as to hang loosely by the side, and let the recruit breathe quietly for one or two respirations before fully expanding and emptying the chest. It is a good plan for the medical examiner to show the recruit what is needed, and to explain to him that by whistling he can more effectually empty his chest.

Passing next to eyesight, the recruit's vision is tested by Snellen's types, that by dots having been done away with. Each eye must be tested separately, and if D-24 can be read at 20 ft. with each eye without glasses, the recruit is considered "fit." Should he fail with either eye with D-24, but can do D-6 with one eye without glasses at 20 ft., and not less than D-36 with the other without glasses, he will be regarded as "fit." When a recruit does not reach either of these standards he must be rejected. Nothing definite is laid down in the instructions about glasses, but if recruits who use glasses come up to D-6 at 20 ft. with each eye they can be accepted.

If the recruit satisfies the medical examiner in all the above respects, his general examination is proceeded with. This is conducted on definite lines, attention being first directed to the physical development, then to the trunk, next to the upper extremities, and lastly to the lower limbs. The heart and lungs are subjected to a careful stethoscopic examination, and then steps are taken to detect any deformities and to be sure that the recruit has full use of all his joints. The medical examiner satisfies himself by the usual tests of the absence of hernia, varicocele, varicose veins, extreme flatness of feet, and that there is no otorrhœa. It is also important to observe carefully the intelligence, character of voice, and power of hearing of the man, and this can be done to a large extent by his replies to the questions put to him.

The condition of the teeth must not be neglected. The rule in the Regular Army is that there must be two molars meeting either on one side or the other; in other words, there must be four double teeth in working order, together with a fairly good set of front teeth. In the Territorial Force it is allowable to pass a recruit if he has two molars in apposition on only one side of four bicuspids, provided it is thought digestion would not be interfered with and he seems well nourished.

The above outline of medical examination should be followed, and it will be found to embrace all that is essentially needed so as to ensure only the admission of men of general fitness, who will be able to stand the strain of field training and meet the extra physical demands of camp life, especially if there is conjoined with this health a sound knowledge of personal hygiene.

